# *Powder X-Ray Reference Patterns of* Sr_2_RGaCu_2_O*_y_* (R = Pr, Nd, Sm, Eu, Gd, Dy, Ho, Er, Tm, *and* Y)

**DOI:** 10.6028/jres.106.032

**Published:** 2001-08-01

**Authors:** W. Wong-Ng, J. A. Kaduk, I. Levin, W. Greenwood, J. Dillingham

**Affiliations:** National Institute of Standards and Technology, Gaithersburg, MD 20899-8520; BP-Amoco Corporation, Naperville, IL 60566-7011; National Institute of Standards and Technology, Gaithersburg, MD 20899-8520; Geology Department, University of Maryland, College Park, MD

**Keywords:** Rietveld refinements, Sr_2_RGaCu_2_O_7_, superconductor related phases, x-ray powder patterns

## Abstract

X-Ray Rietveld refinements were conducted on a series of eleven lanthanide phases, Sr_2_RGaCu_2_O*_y_* (2112 phase, R = Pr, Nd, Sm, Eu, Gd, Dy, Ho, Y, Er, Tm, and Yb) that are structurally related to the high *T*_c_ superconductor Ba_2_YCu_3_O_7_ (213). In the 2112 structure, instead of square planar Cu-O chains, tetrahedral GaO4 chains were found to run in a zig-zag fashion along the diagonal of the basal 213 *ab*-direction. Reference powder patterns for these compounds were prepared by using the Rietveld decomposition technique. The unit cell volume of these compounds follows the expected trend of the lanthanide contraction. The lattice parameters range from *a* = 22.9694(3) Å, *b* = 5.5587(2) Å, and *c* = 5.44743(7) Å for R = Pr, to *a* = 22.8059(2) Å, *b* = 5.46031(5) Å, and *c* = 5.37773(5) Å for R = Yb. An electon diffraction study of the Sm- and Er-analogs showed characteristic diffuse streaks along the *b*-axis, suggesting some disorder within the GaO_4_ chains.

## 1. Introduction

During the search of high *T*_c_ superconductors, various related phases built from the alternate blocks of perovskite (ABO_3−_*_x_*) and rock salt (AO) units were discovered; these compounds have a general formula (AO)*_m_*(ABO_3−_*_x_*)*_n_*. Two such families are the well-known high temperature superconductor Ba_2_RCu_3_O_7_ (213, R = lathanides and yttrium) [1], and also the Sr_2_R(M_3−_*_x_*Cu*_x_*)O*_y_* series [2–5], where R = lanthanides and Y. Superconductivity was observed in some Sr_2_R(M_3−_ *_x_*Cu*_x_*)O*_y_* compounds [2,3]. The structure with *x* = 1 is known as the 2112 type. Detailed structural analysis of these compounds can provide further understanding of the behavior of cuprate superconductors.

**Fig. 1:**
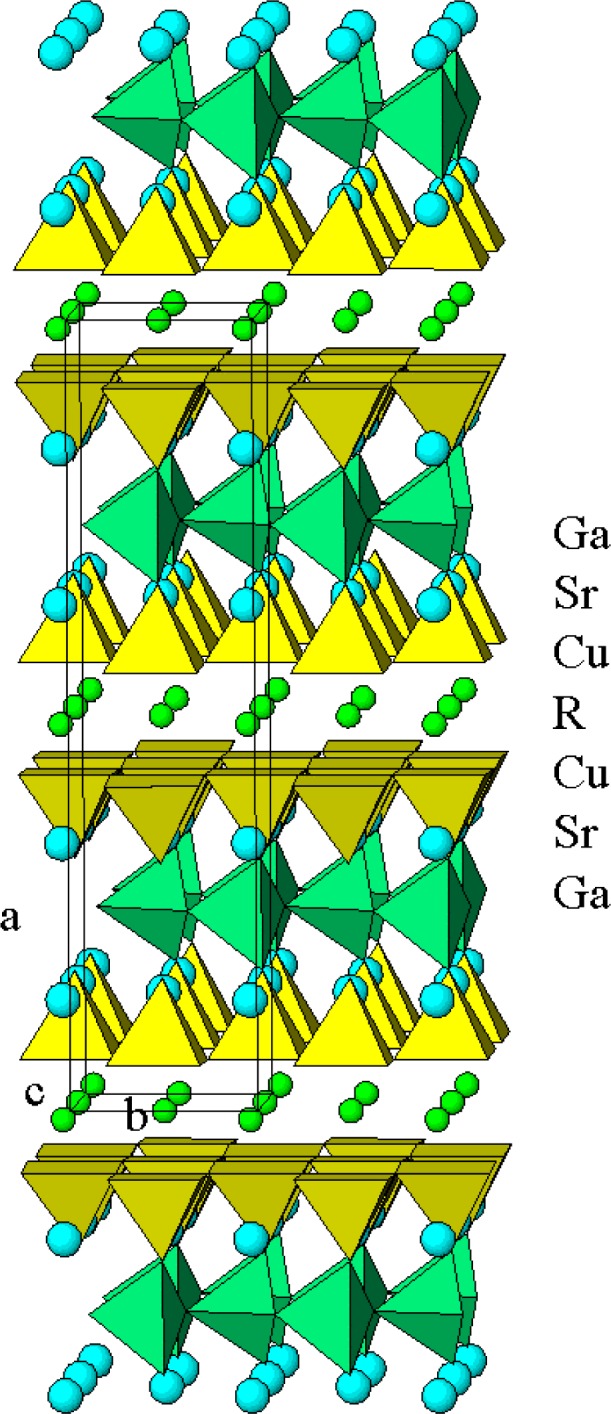
Crystal Structure of Sr_2_RGaCu_2_O*_y_* [5].

Sr_2_RGaCu_2_O*_y_* (Ga-2112) crystallizes in a space group Ima2 [4] with structure related to that of Ba_2_YCu_3_O_7_. Ba_2_YCu_3_O_7_ crystallizes in a space group Pmmm with lattice parameters of *a* = 3.8198(1) Å, *b* = 3.8849(1) Å, and *c* = 11.6762(3) Å [1]. Substitution of one-third of Cu in Sr_2_RCu_3_O_6+_*_y_* by Ga results in the chemical formula of Sr_2_RGaCu_2_O*_y_*. The relationship between the lattice parameters of Sr_2_RGaCu_2_O*_y_* and those of Ba_2_YCu_3_O_7_ was found to be: *a*_(2112)_ ≈ 22.8 Å ≈ 2 · *c*_(213)_, *b*_(2112)_ ≈5.5 Å≈√2 · *b*_(213)_ and *c*_(2112)_ ≈5.4 Å≈√2 · *a*_(213)_. The structure of the 2112 phase (Fig. 1) has been discussed in detail by Vaughey et al. [4] and Roth et al. [5]. In both the 2112 and 213 structures [6], double-layers of corner-sharing CuO_5_ pyramids are separated by the lanthanide ions. In Sr_2_RGaCu_2_O*_y_*, the connection between two double-layers of CuO_5_ along the *a*-axis is mediated by GaO_4_ tetrahedra. Tetrahedral GaO_4_ chains were found running along the *c*-axis (the diagonal of the basal plane of the Ba_2_YCu_3_O_7_ compound) in a zig-zag fashion, which is contrast to the square-planar copper chains typically observed in the 213 compounds. The 8-fold oxygen coordination of R is similar to that found in the 213-phase. Sr plays the role of Ba in 213 by filling the large voids located approximately at the same *x*-coordinates as the apical oxygens of the CuO_5_ units. As a result, Sr has a total of 9-fold coordination.

As the powder x-ray diffraction technique is of primary importance for phase characterization, extensive coverage and accurate reference diffraction patterns of the superconductor and related phases in the Powder Diffraction File (PDF) [7] is essential for the high *T*_c_ superconductivity community. The main goal of this paper is to prepare the standard reference patterns for this series of compounds using the Rietveld refinement method. An electron diffraction study was also carried out to determine if the superlattice lattice exists for the lanthanide Ga-2112 structures.

## 2. Experimental

### 2.1 Sample Preparation

About (1–2) g each of the eleven polycrystalline samples of the Sr_2_RGaCu_2_O*_y_* series (R = Pr, Nd, Sm, Eu, Gd, Dy, Ho, Er, Tm,Y, and Yb) were prepared by the high temperature solid state sintering method. Stoichiometric powders of SrCO_3_, R_2_O_3_ (R = Nd to Lu) or Pr_6_O_11_, Ga_2_O_3_ and CuO were mixed and compacted by pressing the powder in a pelletizing die, and were heat treated in air according to the schedule of 850 °C for 2 d, 960 °C for 5 d and 1000 °C for 8 d. Each time after the samples were taken out of the furnace, they were reground and repelletized. Since the differential thermal analysis (DTA) melting temperatures of the Y- and Nd-analogs take place at 1080 °C and 1130 °C, respectively [5], the highest temperature of sample preparation for most samples is below 1050 °C to avoid melting. The highest temperatures of heat treatment for the Tm, Yb, and Lu compositions were around 975 °C and 980 °C. X-Ray powder diffraction was used to identify the phases synthesized and to confirm phase purity.

### 2.2 Reference Powder X-ray Patterns

#### 2.2.1 Experimental Measurement

For standard pattern measurements, the black Sr_2_RGaCu_2_O*_y_* powders were mounted in zero-background quartz holders with double-sided adhesive tape. A Scintag PAD V diffractometer^1^ equipped with an Ortec intrinsic Ge detector was used to measure the powder patterns (CuKα radiation, 40 KV, 30 mA) from 3°–140° 2θ in 0.02° steps every 10 s.

#### 2.2.2 Patterns Analysis

All data processing was carried out using the Rietveld structural refinement technique [8] with the computer program suite GSAS [9]. Published structural models were used [4,5]. A scale factor, a sample displacement coefficient, the atomic coordinates, isotropic displacement coefficients, and the orthorhombic lattice parameters were refined. The diffraction peak profiles were described using a pseudo-Voigt function; only the Gaussian W and Cauchy X (size) terms were refined. Background intensities were described using a 3-term cosine Fourier series.

Reference x-ray patterns of the 10 Sr_2_RGaCu_2_O*_y_* compounds, where R = Pr, Nd, Sm, Eu, Gd, Dy, Ho, Er, Tm, and Y were obtained with a Rietveld pattern decomposition technique. These patterns represent ideal specimen patterns. They are corrected for systematic errors both in *d*-spacings and intensity *I*. The reported peak positions are calculated from the refined lattice parameters, as they represent the best measure of the true positions. For peaks resolved at the instrument resolution function, the individual peak positions are reported. For overlapping peaks, the intensity-weighted average peak position is reported with multiple indices. For marginally-resolved peaks, individual peaks are reported to simulate the visual appearance of the pattern.

#### 2.2.3 Electron Diffraction Studies

Electron diffraction patterns were measured for the R = Sm and R = Er samples to ensure that a superlattice does not exist in these compounds. The specimens were prepared from the sintered pellets by conventional grinding, polishing and ion thinning. The specimens were examined using a Phillips 430 TEM operated at 200 kV.

## 3. Results and Discussion

All samples except the Tm and Yb compounds were confirmed to contain a single phase. A small quantity of Yb_2_Cu_2_O_5_, SrYb_2_O_4_ and GaCuO_2_ were found to coexist with the Sr_2_YbGaCu_2_O*_y_* phase. The pattern for the Yb-analog was not measured because of impurities in the powder. In addition, the smaller size Lu analog cannot be prepared even at a relatively high temperature of 1050° C. Rather, an x-ray diffraction pattern of a specimen with a nominal composition of Sr_2_LuGaCu_2_O*_y_* clearly showed a mixture of Lu_2_Cu_2_O_5_, (Sr,Lu)_14_Cu_24_O_41_, and Sr_4_Ga_2_O_7_, etc. Apparently, the Lu^3+^ ion is too small for the 8-fold oxygen coordination cage; therefore, the compound Sr_2_LuGaCu_2_O*_y_* is unstable.

**Fig. 2:**
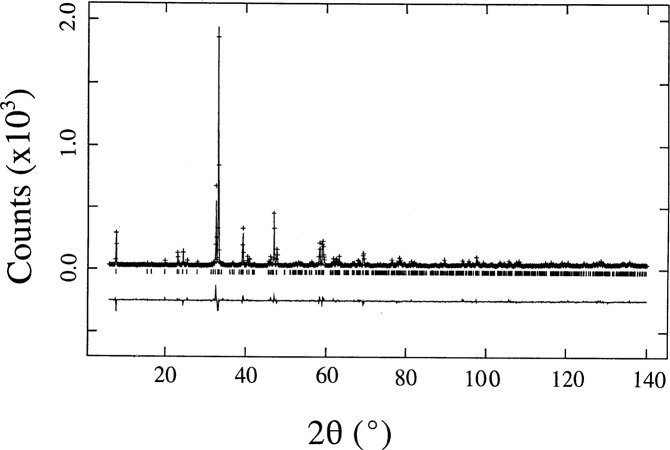
Observed, calculated, and difference x-ray Rietveld powder patterns of Sr_2_EuGaCu_2_O*_y_*. The crosses represent the observed data points, and the solid line represent the calculated pattern. The difference pattern is plotted at the same scale as the other patterns. The row of tick marks indicates the calculated peak positions.

**Fig. 3:**
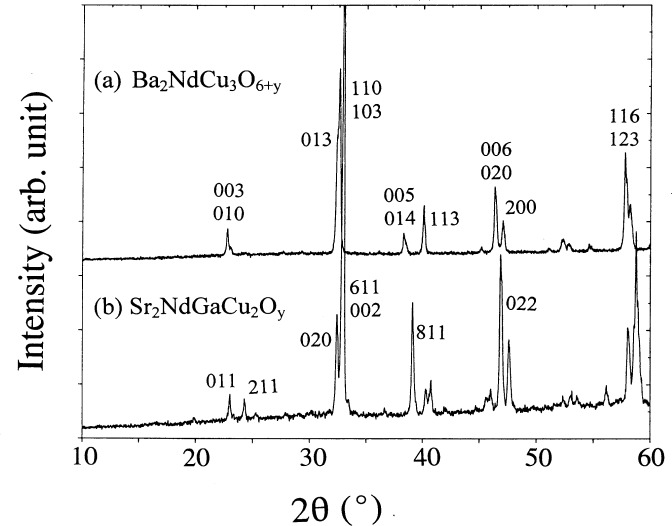
X-Ray diffraction patterns of Sr_2_NdGaCu_2_O*_y_* and Ba_2_NdCu_3_O_6+_*_y_*. Similarity of these patterns is discernible. Representative *hkl* values are indicated.

**Fig. 4:**
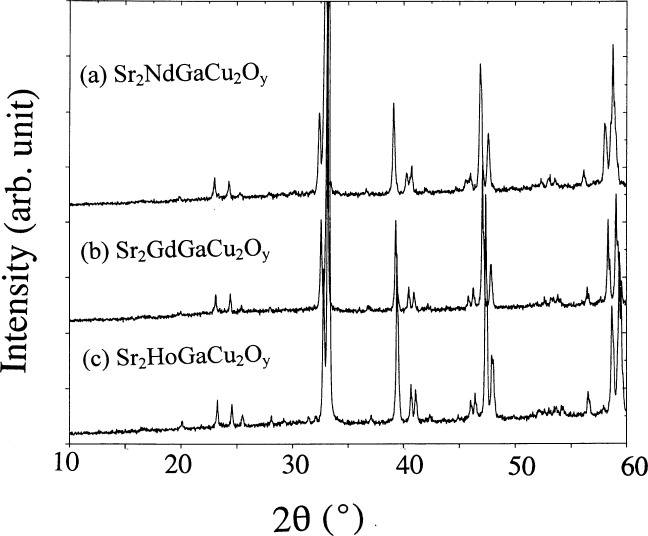
X-Ray diffraction patterns of three selected samples (Sr_2_RGaCu_2_O*_y_*, R = Nd, Gd and Ho).

The Rietveld refinement results in an acceptable fit to the experimental data (Fig. 2). The similarity of both Sr_2_NdGaCu_2_O*_y_* and Ba_2_NdCu_3_O_6+_*_y_* structures is revealed in the similarity of their x-ray powder patterns (Fig. 3). X-ray diffraction patterns of three selected samples (Sr_2_RGaCu_2_O*_y_*, R = Nd, Gd, and Ho) are shown in Fig. 4; as expected, the patterns of these analogs are similar up to the small displacements of the corresponding reflections.

**Fig. 5:**
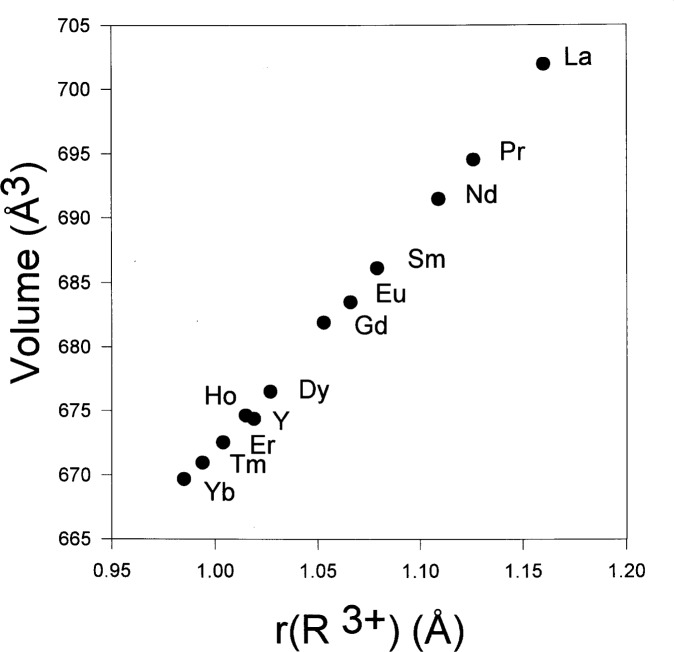
Plots of the unit cell volumes of Sr_2_NdGaCu_2_O*_y_* vs ionic radius of R^3+^, r(R^3+^) [10,11]. A general trend parallel to the lanthanide contraction is shown.

X-ray powder diffraction showed the structure of SR_2_RGaCu_2_O*_y_* to be Ima2. The lattice parameters, densities, and ionic radii [10,11] of these phases are listed in Table 1. The lattice parameters of Sr_2_RGaCu_2_O*_y_* range from *a* = 23.129(1) Å, *b* = 5.5587(2) Å, and *c* = 5.4596(3) Å for R = La [12], to *a* = 22.7964(3) Å for R = Er, and *b* = 5.46031(5) Å, and *c* = 5.37773(5) Å for R = Yb. The numbers in parentheses indicate the standard uncertainties, Type A, calculated by the GSAS program suite [9]. Fig. 5 shows a dependence of the unit cell volume on the ionic radius *r* (R^3+^) of R = La, Pr, Nd, Sm, Eu, Gd, Dy, Ho, Y, Er, Tm, and Yb. Except for Ho, a monotonic decrease in volume on going from La to Yb is observed.

**Fig. 6:**
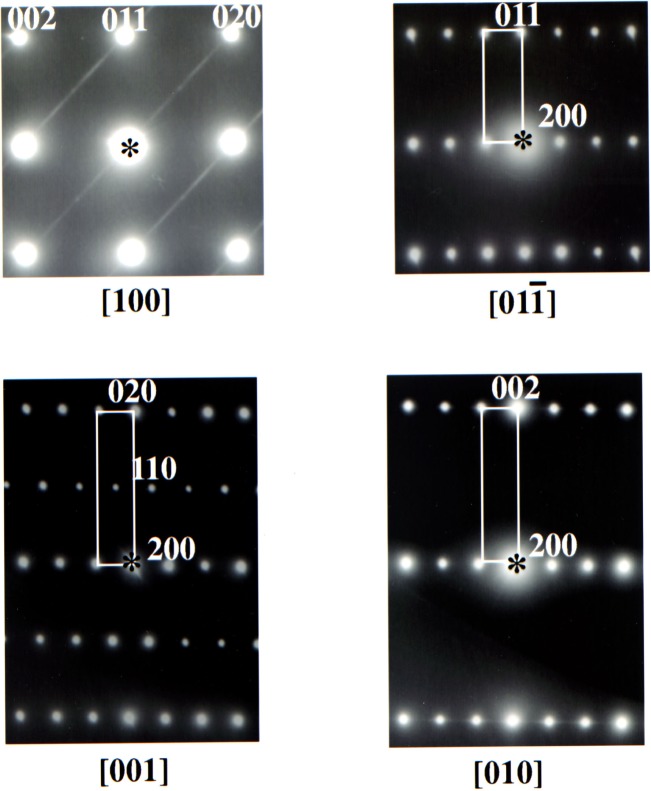
Selected area (electron) diffraction (SAD) patterns of Sr_2_SmGaCu_2_O*_y_* showing various zone-axis orientations. Continuous streaks of diffuse intensity along the *b*-direction is observed in the [100] zone-axis pattern.

In the 2112 structure, instead of square planar Cu-O chains, tetrahedral GaO_4_ chains were found to run in a zig-zag fashion along the diagonal of the basal 213 *ab*-direction. A set of selected area (electron) diffraction (SAD) patterns for the R = Sm sample is illustrated in Fig. 6. In addition to the strong fundamental reflections, the pattern of the [100] zone axis exhibits continuous streaks of diffuse intensity along the *b*-direction. Formation of a superstructure with a doubled periodicity along the *c*-direction has been reported for the Sr_2_YCoCu_2_O_7_ and Sr_2_ (Nd,Ce)_2_GaCu_2_O_9_ compounds by Krekels et al. [13]. However, in the present study, no such doubling was observed. The observed streaks can be attributed to a disorder due to presence of oxygen vacancies and/or copper atoms within tetrahedral GaO_4_ chains extending along the *b*-direction, similar to that described by Krekels et al. [12]. X-ray diffraction, however, is not as sensitive as neutron diffraction to the disorder of light elements such as oxygen, as reflected in relatively small values (≈ 0.004 Å) of the refined isotropic temperature factors (*U*_iso_).

Tables 2–11 list the reference patterns for Sr_2_RGaCu_2_O*_y_* (R = Pr, Nd, Sm, Eu, Gd, Dy, Ho, Er, Tm, and Y). The tables present the *d*-spacing values, Miller indices and integrated intensities which were normalized to a maximum value of 999. The symbols “M” and “+” refer to peaks containing two or more than two reflections, respectively.

## 4. Summary

X-ray diffraction patterns of the Sr_2_RGaCu_2_O*_y_* phases were prepared for R = Pr, Nd, Sm, Eu, Gd, Dy, Ho, Er, Tm, and Y. These patterns are similar to those of the well known high-temperature superconductors Ba_2_YCu_3_O_6+_*_y_*. The Lu analog could not be prepared even using higher temperature and prolonged heat-treatments, possibly due to a small size of Lu^3+^, which makes it unstable in the 8-fold coordination.

X-ray powder diffraction showed the structure of SR_2_RGaCu_2_O*_y_* to be Ima2. GaO_4_ tetrahedral chains were found along the diagonal base of the 213-type cell. Electron diffraction study revealed continuous streaks of diffuse intensity attributed to the presence of oxygen vacancy disorder, and/or the presence of a Cu atom within the GaO_4_ chains. A neutron diffraction study will be conducted to investigate the possible presence of the disordered chains.

## Figures and Tables

**Table 1:** Crystallographic data for the Sr_2_RGaCu_2_O*_y_* series (Ima2, *Z* = 4; R = La, Pr, Nd, Sm, Eu, Gd, Dy, Y, Ho, Er, Tm, and Yb). The effective ionic radii of R^3+^ (eight-coordination) were taken from Shannon’s Table [10,11]. D_c_ refers to calculated density

R	R^3+^ Å	*a* (Å)	*b* (Å)	*c* (Å)	Volume (Å^3^)	*D*_c_(kg/m^2^)
La	1.160	23.129(1)	5.5587(2)	5.4596(3)	701.93	5.895 [12]
Pr	1.126	22.9694(3)	5.55081(7)	5.44743(7)	694.54	5.977
Nd	1.109	22.9206(2)	5.54344(5)	5.44229(5)	691.49	6.035
Sm	1.079	22.8708(2)	5.52690(5)	5.42790(5)	686.11	6.147
Eu	1.066	22.8441(3)	5.51870(8)	5.42102(8)	683.47	6.181
Gd	1.053	22.8294(2)	5.51236(5)	5.41835(5)	681.87	6.247
Dy	1.027	22.8015(2)	5.49286(5)	5.40138(5)	676.50	6.348
Y	1.019	22.8113(2)	5.48069(5)	5.39397(5)	674.36	5.643
Ho	1.015	22.7975(2)	5.48433(5)	5.39562(5)	674.61	6.390
Er	1.004	22.7964(3)	5.47497(6)	5.38837(6)	672.52	6.433
Tm	0.994	22.8024(3)	5.46762(7)	5.38158(7)	670.95	6.464
Yb	0.985	22.8059(2)	5.46031(5)	5.37773(5)	669.67	6.554

**Table 2:** X-Ray reference pattern for Sr_2_PrGaCu_2_O*_y_* (space group Ima2, *Z* = 4, *a* = 22.9694(3) Å, *b* = 5.55081(7) Å, and *c* = 5.44743(7) Å)

*d*	*I*	*h*	*k*	*l*	*d*	*I*	*h*	*k*	*l*	*d*	*I*	*h*	*k*	*l*
11.4847	91	2	0	0	5.39550	6	1	1	0	4.49403	20	3	1	0
3.88795	59	0	1	1	3.82823	6	6	0	0	3.68265	47	2	1	1
3.53906	21	5	1	0	3.21943	11	4	1	1	2.87117	11	8	0	0
2.82470	16	7	1	0	2.77541191		0	2	0	2.72689999*		6	1	1M
2.72689999*		0	0	2M	2.69775	5	2	2	0	2.65020	10	2	0	2
2.49884	5	4	2	0	2.45922	14	4	0	2M	2.45922	14	1	2	1M
2.30965179		8	1	1	2.24701	50	6	2	0	2.21932	40	6	0	2
2.15848	7	5	1	2	1.99551	22	8	2	0	1.97617	29	8	0	2+
1.94397207		0	2	2	1.91671	26	2	2	2	1.91412	87	12	0	0
1.84132	8	4	2	2	1.77598	7	9	2	1	1.75197	10	0	3	1
1.73330	9	6	2	2	1.72582	6	0	1	3	1.71629	11	5	3	0
1.70665	5	2	1	3	1.65278	5	4	1	3	1.64067	27	14	0	0
1.61078	11	7	3	0M	1.61078	11	8	2	2M	1.59307129		6	3	1
1.57571	39	12	2	0	1.57333157		6	1	3	1.56607	53	12	0	2
1.51618	7	1	2	3	1.51159	43	14	1	1	1.49553	46	8	3	1
1.47917	34	8	1	3	1.45205	10	5	3	2	1.41235	21	14	2	0
1.40539	11	14	0	2	1.38757	26	0	4	0M	1.38757	26	7	3	2
1.37768	10	2	4	0	1.36392	75	12	2	2	1.36186	29	0	0	4
1.34246	5	1	4	1	1.29598	5	0	3	3	1.25381	36	14	2	2
1.23647	16	0	4	2	1.22937	5	2	4	2	1.22755	40	6	3	3
1.22260	25	0	2	4	1.21244	15	18	1	1	1.19754	10	14	3	1
1.18923	23	9	4	1M	1.18923	23	14	1	3M	1.18123	20	8	3	3
1.17662	14	6	4	2	1.16465	10	6	2	4	1.12351	12	12	4	0
1.10966	14	12	0	4	1.10142	39	20	1	1	1.08780	5	0	5	1
1.06120	6	20	2	0	1.05953	16	14	4	0	1.05824	9	20	0	2
1.04789	12	14	0	4	1.04638	23	6	5	1	1.03862	13	12	4	2
1.03147	10	18	3	1	1.02991	58	12	2	4M	1.02991	58	6	1	5M
1.02606	11	18	1	3	1.01716	25	8	5	1M	1.01716	25	14	3	3M
1.00323	6	5	5	2	1.00189	11	8	1	5	0.98880	7	20	2	2
0.98745	19	14	4	2	0.98034	17	14	2	4	0.97199	17	0	4	4
0.96852	5	2	4	4	0.96049	36	20	3	1	0.95611	26	20	1	3
0.94717	7	0	5	3	0.91944	21	6	5	3	0.91180	27	6	3	5
0.90928	7	18	3	3	0.90790	9	0	0	6	0.89942	23	8	5	3M
0.89942	23	6	6	0M	0.89571	10	14	1	5	0.89233	14	8	3	5
0.88342	11	26	0	0M	0.88342	11	6	0	6M	0.87796	6	20	0	4
0.87598	15	0	6	2	0.87345	9	2	6	2	0.86665	24	12	4	4
0.86291	28	0	2	6	0.86148	23	26	1	1	0.86048	6	2	2	6
0.85953	49	20	3	3	0.85875	10	9	6	1M	0.85875	10	24	2	2M
0.85391	6	6	6	2	0.84874	11	12	5	3M	0.84874	11	21	4	1M
0.84171	30	26	2	0M	0.84171	30	20	4	2M	0.84034	14	26	0	2
0.83705	26	20	2	4M	0.83705	26	24	1	3M	0.83620	43	14	4	4M
0.83620	43	11	6	1M	0.83295	5	12	6	0	0.82783	9	18	5	1
0.82030	26	12	0	6M	0.82030	26	14	5	3M					

**Table 3:** X-Ray reference pattern for Sr_2_NdGaCu_2_O*_y_* (space group Ima2, *Z* = 4, *a* = 22.9206(2) Å, *b* = 5.54344(5) Å, and *c* = 5.44229(5) Å)

*d*	*I*	*h*	*k*	*l*	*d*	*I*	*h*	*k*	*l*	*d*	*I*	*h*	*k*	*l*
11.4603145		2	0	0	4.48683	15	3	1	0	3.88355	68	0	1	1
3.82010	6	6	0	0	3.67810	45	2	1	1	3.53269	18	5	1	0
3.21478	6	4	1	1	2.86508	8	8	0	0	2.81928	16	7	1	0
2.77172209		0	2	0	2.72286999*		6	1	1M	2.72286999*		0	0	2M
2.69405	6	2	2	0	2.64754	9	2	0	2	2.45564	8	1	2	1
2.30555212		8	1	1	2.29206	5	10	0	0	2.24341	57	6	2	0
2.21634	53	6	0	2	2.15576	7	5	1	2	1.99209	24	8	2	0
1.94177234		0	2	2	1.91449	27	2	2	2	1.91005	93	12	0	0
1.77301	8	9	2	1	1.75304	5	10	0	2	1.74971	12	0	3	1
1.73099	10	6	2	2	1.72412	9	0	1	3	1.71382	13	5	3	0
1.70494	5	2	1	3	1.63719	26	14	0	0	1.60838	11	7	3	0M
1.60838	11	8	2	2M	1.59078153		6	3	1	1.57174191		12	2	0M
1.57174191		6	1	3M	1.56336	52	12	0	2	1.51457	8	1	2	3
1.50861	48	14	1	1	1.49327	49	8	3	1	1.47727	44	8	1	3
1.45017	13	5	3	2	1.40964	32	14	2	0	1.40285	19	14	0	2
1.38571	25	0	4	0M	1.38571	25	7	3	2M	1.37584	9	2	4	0
1.36138	95	12	2	2M	1.36138	95	0	0	4M	1.35108	6	2	0	4
1.34402	6	16	1	1	1.34070	6	1	4	1	1.29452	5	0	3	3
1.25166	43	14	2	2	1.23493	23	0	4	2	1.22782	6	2	4	2
1.22603	50	6	3	3	1.22136	30	0	2	4	1.20999	17	18	1	1
1.19547	12	14	3	1	1.18737	20	9	4	1M	1.18737	20	14	1	3M
1.17969	18	8	3	3	1.17505	15	6	4	2	1.16335	11	6	2	4
1.12353	5	8	2	4	1.12171	13	12	4	0	1.10817	18	12	0	4
1.09917	46	20	1	1	1.08637	5	0	5	1	1.05907	8	20	2	0
1.05777	22	14	4	0	1.05618	8	20	0	2	1.04640	15	14	0	4
1.04494	32	6	5	1	1.03705	15	12	4	2	1.02873	60	12	2	4M
1.02873	60	6	1	5M	1.02429	10	18	1	3	1.01568	29	8	5	1M
1.01568	29	14	3	3	1.00079	13	8	1	5	0.98696	7	20	2	2
0.98590	23	14	4	2	0.97896	20	14	2	4	0.97089	13	0	4	4
0.95869	29	20	3	1	0.95442	30	20	1	3	0.91827	22	6	5	3
0.91080	27	6	3	5	0.90779	6	18	3	3	0.90705	6	0	0	6
0.89823	23	8	5	3	0.89823	23	6	6	0	0.89454	9	14	1	5
0.89131	15	8	3	5	0.88251	6	6	0	6	0.88156	5	26	0	0
0.87485	14	0	6	2	0.87232	10	2	6	2	0.86549	25	12	4	4
0.86206	21	0	2	6	0.85967	23	26	1	1M	0.85967	23	2	2	6M
0.85808	39	20	3	3	0.85278	7	6	6	2	0.84773	6	12	5	3
0.84701	6	21	4	1	0.84092	7	6	2	6	0.84007	26	26	2	0M
0.84007	26	20	4	2M	0.83865	15	26	0	2	0.83573	19	20	2	4
0.83509	34	14	4	4	0.83461	5	11	6	1	0.83172	6	12	6	0
0.82647	8	18	5	1										

**Table 4:** X-Ray reference pattern for Sr_2_SmGaCu_2_O*_y_* (space group Ima2, *Z* = 4, *a* = 22.8708(2) Å, *b* = 5.52690(5) Å, and *c* = 5.42790(5) Å)

*d*	*I*	*h*	*k*	*l*	*d*	*I*	*h*	*k*	*l*	*d*	*I*	*h*	*k*	*l*
11.4356213		2	0	0	5.71781	8	4	0	0	5.37248	6	1	1	0
4.47485	14	3	1	0	3.87263	56	0	1	1	3.81187	6	6	0	0
3.66801	60	2	1	1	3.52395	20	5	1	0	3.20641	6	4	1	1
2.85891	6	8	0	0	2.81264	12	7	1	0	2.76356242		0	2	0
2.71613999*		6	1	1M	2.71613999*		0	0	2M	2.64051	5	2	0	2
2.44856	5	1	2	1	2.300051	86	8	1	1	2.28712	23	10	0	0
2.23742	43	6	2	0	2.21079	31	6	0	2	2.15014	7	5	1	2
1.96932	21	10	1	1	1.93632220		0	2	2	1.90620119		2	2	2M
1.90620119		12	0	0M	1.76852	7	9	2	1	1.72551	14	6	2	2M
1.72551	14	2	3	1M	1.70958	13	12	1	1M	1.70958	13	5	3	0M
1.70034	7	2	1	3	1.63366	18	14	0	0	1.60482	5	7	3	0
1.58871	5	11	2	1	1.586361	25	6	3	1	1.56898	34	12	2	0
1.56737157		6	1	3	1.55972	49	12	0	2	1.51039	6	1	2	3
1.50521	41	14	1	1	1.48922	44	8	3	1	1.48471	5	3	2	3
1.47782	6	10	2	2	1.47348	31	8	1	3	1.46985	5	15	1	0
1.44612	9	5	3	2	1.40632	29	14	2	0	1.39963	12	14	0	2
1.38162	40	0	4	0M	1.38162	40	7	3	2M	1.37180	5	2	4	0
1.35831	70	12	2	2	1.35692	23	0	0	4	1.34747	5	2	0	4
1.34101	9	16	1	1	1.29853	5	3	1	4	1.28689	5	12	3	1
1.24863	45	14	2	2	1.23136	14	0	4	2	1.22267	48	6	3	3
1.21802	24	0	2	4	1.20730	19	18	1	1	1.20376	5	4	4	2
1.19247	7	14	3	1	1.18455	18	9	4	1M	1.18455	18	14	1	3
1.17650	18	8	3	3	1.17174	10	6	4	2	1.16023	13	6	2	4
1.11871	16	12	4	0	1.10553	13	16	3	1M	1.10553	13	12	0	4M
1.09676	41	1	4	3M	1.09676	41	20	1	1M	1.05667	8	20	2	0
1.05501	21	14	4	0	1.05382	8	20	0	2	1.04382	13	14	0	4
1.04194	25	6	5	1	1.03428	13	12	4	2	1.02618	69	12	2	4+
1.02618	69	6	1	5+	1.02188	8	18	1	3	1.01292	23	8	5	1
0.99904	5	5	5	2	0.99816	13	8	1	5	0.98466	10	20	2	2
0.98332	18	14	4	2	0.97648	13	14	2	4	0.96816	20	0	4	4
0.96560	8	10	1	5	0.95641	30	20	3	1	0.95457	5	4	4	4
0.95220	29	20	1	3	0.94329	5	0	5	3	0.93530	5	18	4	0
0.93294	5	16	4	2	0.91574	23	22	2	2M	0.91574	23	6	5	3M
0.90832	31	6	3	5	0.90461	10	0	0	6	0.89579	18	8	5	3
0.89521	5	19	4	1	0.89350	7	4	0	6	0.89227	5	14	1	5
0.88915	17	22	1	3M	0.88915	17	8	3	5M	0.88017	6	6	0	6
0.87215	20	0	6	2M	0.87215	20	10	5	3M	0.86978	5	2	6	2
0.86568	5	10	3	5	0.86318	12	12	4	4	0.86233	7	4	6	2
0.85973	26	0	2	6	0.85781	18	26	1	1	0.85599	48	20	3	3
0.85523	8	9	6	1	0.85413	10	16	1	5	0.85303	5	1	4	5
0.85017	7	4	2	6	0.84493	11	21	4	1	0.83822	33	26	2	0
0.83656	27	26	0	2M	0.83656	27	24	3	1M	0.83351	22	24	1	3
0.83288	27	14	4	4	0.83227	8	11	6	1	0.82939	7	12	6	0
0.82524	6	22	0	4	0.82431	7	18	5	1	0.82038	5	1	6	3

**Table 5:** X-Ray reference pattern for Sr_2_EuGaCu_2_O*_y_* (space group Ima2, *Z* = 4, *a* = 22.8441(3) Å, *b* = 5.51870(8) Å, and *c* = 5.42102(8) Å)

*d*	*I*	*h*	*k*	*l*	*d*	*I*	*h*	*k*	*l*	*d*	*I*	*h*	*k*	*l*
11.4221175		2	0	0	5.71104	5	4	0	0	4.46854	16	3	1	0
3.86731	55	0	1	1	3.80736	7	6	0	0	3.66305	53	2	1	1
3.51929	21	5	1	0	3.20220	7	4	1	1	2.85552	7	8	0	0
2.80905	11	7	1	0	2.75935347		0	2	0	2.71251999*		6	1	1M
2.71251999*		0	0	2M	2.63727	12	2	0	2	2.44499	10	1	2	1
2.297171	91	8	1	1	2.28441	10	10	0	0	2.23427	47	6	2	0
2.20811	42	6	0	2	2.14742	6	5	1	2	1.98428	21	8	2	0
1.96589	29	8	0	2	1.93366275		0	2	2	1.90418	98	2	2	2M
1.90418	98	12	0	0M	1.78813	5	3	3	0	1.76617	10	9	2	1
1.75964	5	10	2	0	1.74678	6	10	0	2	1.74200	10	0	3	1
1.72405	12	6	2	2	1.71729	10	0	1	3	1.70644	10	5	3	0
1.69821	6	2	1	3	1.64455	5	4	1	3	1.63172	22	14	0	0
1.60110	5	8	2	2	1.58407142		6	3	1	1.56577188		12	2	0M
1.56577188		6	1	3M	1.55785	58	12	0	2	1.50840	5	1	2	3
1.50338	46	14	1	1	1.48712	48	8	3	1	1.47591	7	10	2	2
1.47166	50	8	1	3	1.44409	8	5	3	2	1.40453	17	14	2	0
1.39796	16	14	0	2	1.37963	37	0	4	0M	1.37963	37	7	3	2M
1.36972	7	2	4	0	1.35617	92	12	2	2M	1.35617	92	0	0	4M
1.33939	10	16	1	1	1.33477	5	1	4	1	1.29714	5	6	4	0
1.28910	5	0	3	3	1.24705	37	14	2	2	1.22956	22	0	4	2
1.22102	47	6	3	3	1.21645	29	0	2	4	1.20585	18	18	1	1
1.19088	12	14	3	1	1.18290	18	14	1	3	1.17493	21	8	3	3
1.17006	11	6	4	2	1.15875	10	6	2	4	1.11714	9	12	4	0
1.10405	12	12	0	4	1.09543	32	20	1	1	1.05536	8	20	2	0
1.05355	18	14	4	0	1.05257	11	20	0	2	1.04255	12	14	0	4
1.04039	27	6	5	1	1.03285	14	12	4	2	1.02491	61	12	2	4+
1.02491	61	6	1	5+	1.02065	9	18	1	3	1.01146	24	14	3	3M
1.01146	24	8	5	1M	0.99711	18	5	5	2M	0.99711	18	8	1	5M
0.98345	10	20	2	2	0.98198	23	14	4	2	0.97526	16	14	2	4
0.96683	24	0	4	4	0.95519	30	20	3	1	0.95105	28	20	1	3
0.91978	8	0	6	0	0.91436	23	6	5	3	0.90715	26	6	3	5
0.90439	5	18	3	3	0.90350	6	0	0	6	0.90150	5	14	5	1
0.89440	29	8	5	3M	0.89440	29	6	6	0M	0.89118	10	14	1	5
0.88776	18	8	3	5	0.87909	5	6	0	6	0.87100	16	0	6	2
0.86848	10	2	6	2	0.86203	21	12	4	4	0.85865	20	0	2	6
0.85679	20	26	1	1	0.85623	5	2	2	6	0.85490	43	20	3	3
0.84907	9	6	6	2	0.83722	34	26	2	0+	0.83581	13	26	0	2
0.83267	17	20	2	4	0.83178	28	14	4	4	0.82318	7	18	5	1

**Table 6:** X-Ray reference pattern for Sr_2_GdGaCu_2_O*_y_* (space group Ima2, *Z* = 4, *a* = 22.8294(2) Å, *b* = 5.51236(5) Å, and *c* = 5.41835(5) Å)

*d*	*I*	*h*	*k*	*l*	*d*	*I*	*h*	*k*	*l*	*d*	*I*	*h*	*k*	*l*
11.4147208		2	0	0	4.46419	12	3	1	0	3.86416	57	0	1	1
3.80491	7	6	0	0	3.66013	49	2	1	1	3.51630	20	5	1	0
3.19976	6	4	1	1	2.85368	6	8	0	0	2.80688	13	7	1	0
2.75618209		0	2	0	2.71072999*		6	1	1M	2.71072999*		0	0	2M
2.67919	5	2	2	0	2.63595	14	2	0	2	2.44252	8	1	2	1
2.29555189		8	1	1	2.28294	16	10	0	0	2.23210	46	6	2	0
2.20691	36	6	0	2	2.14608	5	5	1	2	1.98249	20	8	2	0
1.96432	29	8	0	2M	1.96432	29	7	2	1M	1.93208237		0	2	2
1.90315	94	2	2	2M	1.90315	94	12	0	0M	1.76469	6	9	2	1
1.74012	10	0	3	1	1.72271	12	6	2	2	1.71634	7	0	1	3
1.70460	10	5	3	0	1.69726	6	2	1	3	1.63067	19	14	0	0
1.60086	7	7	3	0	1.58248120		6	3	1	1.56478174		12	2	0M
1.56478174		6	1	3M	1.55692	43	12	0	2	1.50736	5	1	2	3
1.50238	39	14	1	1	1.48569	43	8	3	1	1.47481	8	10	2	2
1.47081	45	8	1	3	1.44277	10	5	3	2	1.40344	17	14	2	0
1.39711	15	14	0	2	1.37809	17	0	4	0	1.36816	8	2	4	0
1.35527	81	12	2	2M	1.35527	81	0	0	4M	1.33851	8	16	1	1
1.28805	5	0	3	3	1.24616	42	14	2	2	1.22831	17	0	4	2
1.22015	58	6	3	3+	1.21570	29	0	2	4	1.20505	11	18	1	1
1.18987	8	14	3	1	1.18209	19	14	1	3M	1.18209	19	9	4	1
1.17400	20	8	3	3	1.16891	12	6	4	2	1.15803	9	6	2	4
1.11605	7	12	4	0	1.10345	11	12	0	4	1.09471	42	20	1	1
1.05461	6	20	2	0	1.05237	28	14	4	0M	1.05237	28	20	0	2
1.04198	16	14	0	4	1.03926	26	6	5	1	1.03192	12	12	4	2
1.02455	16	18	3	1M	1.02455	16	12	2	4M	1.02408	36	6	1	5
1.02002	7	18	1	3	1.01048	20	14	3	3M	1.01048	20	8	5	1
0.99640	12	8	1	5	0.98277	7	20	2	2	0.98111	23	14	4	2
0.97465	13	14	2	4	0.96604	18	0	4	4	0.96260	5	2	4	4
0.95445	28	20	3	1	0.95047	32	20	1	3	0.91349	16	6	5	3
0.90655	27	6	3	5	0.90335	12	18	3	3M	0.90335	12	0	0	6M
0.89368	15	8	5	3	0.89306	6	6	6	0	0.89069	7	14	1	5
0.88717	12	8	3	5	0.87865	6	6	0	6	0.87006	11	0	6	2
0.86754	10	2	6	2	0.86135	16	12	4	4	0.85817	18	0	2	6
0.85623	16	26	1	1	0.85429	33	20	3	3	0.84817	7	6	6	2
0.83661	32	26	2	0+	0.83528	14	26	0	2	0.83215	13	20	2	4
0.83114	33	14	4	4	0.82731	5	12	6	0	0.82242	5	18	5	1

**Table 7:** X-Ray reference pattern for Sr_2_DyGaCu_2_O*_y_* (space group Ima2, *Z* = 4, *a* = 22.8015(2) Å, *b* = 5.49286(5) Å, and *c* = 5.40138(5) Å)

*d*	*I*	*h*	*k*	*l*	*d*	*I*	*h*	*k*	*l*	*d*	*I*	*h*	*k*	*l*
11.4008192		2	0	0	4.45195	17	3	1	0	3.85129	75	0	1	1
3.80025	6	6	0	0	3.64872	57	2	1	1	3.50868	18	5	1	0
3.19122	10	4	1	1	2.85019	10	8	0	0	2.80176	15	7	1	0
2.74643209		0	2	0	2.70407999*		6	1	1M	2.70407999*		0	0	2M
2.67005	6	2	2	0	2.62796	17	2	0	2	2.43414	10	1	2	1
2.33024	5	3	2	1	2.29103203		8	1	1	2.28015	16	10	0	0
2.22597	43	6	2	0	2.20141	34	6	0	2	2.14013	5	5	1	2
1.97768	17	8	2	0	1.96040	24	8	0	2	1.92564237		0	2	2
1.89969	92	12	0	0M	1.89969	92	2	2	2M	1.78003	5	3	3	0
1.76050	6	9	2	1	1.74224	5	10	0	2	1.73404	13	0	3	1
1.71771	10	6	2	2	1.71089	7	0	1	3	1.69912	10	5	3	0
1.69195	6	2	1	3	1.63868	5	4	1	3	1.62868	26	14	0	0
1.59609	7	7	3	0	1.58196	5	11	2	1	1.57757	161	6	3	1
1.56260	46	12	2	0	1.560081	47	6	1	3	1.55403	51	12	0	2
1.50247	6	1	2	3	1.50006	34	14	1	1	1.48141	53	8	3	1
1.47119	11	10	2	2	1.46690	47	8	1	3	1.43817	10	5	3	2
1.40088	18	14	2	0	1.39469	17	14	0	2	1.37339	34	7	3	2M
1.37339	24	0	4	0M	1.36336	10	2	4	0	1.35252	49	12	2	2
1.35034	27	0	0	4	1.34097	5	2	0	4	1.33653	8	16	1	1
1.24354	41	14	2	2	1.22407	17	0	4	2	1.21707	5	2	4	2
1.21624	41	6	3	3	1.21179	18	0	2	4	1.20333	12	18	1	1
1.18715	9	14	3	1	1.17964	12	14	1	3	1.17051	21	8	3	3
1.16512	16	6	4	2	1.15452	9	6	2	4	1.10070	10	12	0	4
1.09318	29	20	1	1	1.05296	5	20	2	0	1.05032	5	20	0	2
1.04985	15	14	4	0	1.03952	20	14	0	4	1.03577	30	6	5	1
1.02903	13	12	4	2	1.02288	6	18	3	1	1.02123	50	12	2	4M
1.02123	50	6	1	5M	1.01807	9	18	1	3	1.00822	6	14	3	3
1.00709	14	8	5	1	0.99349	16	8	1	5	0.98103	6	20	2	2
0.97852	20	14	4	2	0.97221	24	14	2	4	0.96282	18	0	4	4
0.95262	32	20	3	1	0.94873	23	20	1	3	0.91048	31	6	5	3
0.90372	26	6	3	5	0.90023	9	0	0	6	0.89081	17	8	5	3
0.88839	6	14	1	5	0.88447	16	8	3	5	0.87698	6	26	0	0
0.87599	5	6	0	6	0.86702	10	0	6	2	0.86452	17	2	6	2
0.85886	20	12	4	4	0.85535	37	0	2	6M	0.85535	37	26	1	1M
0.85253	38	20	3	3+	0.83542	15	26	2	0	0.83427	33	20	4	2+
0.83041	28	24	1	3M	0.83041	28	20	2	4M	0.82883	40	14	4	4
0.82474	7	12	6	0	0.82032	6	18	5	1					

**Table 8:** X-Ray reference pattern for Sr_2_HoGaCu_2_O*_y_* (space group Ima2, *Z* = 4, *a* = 22.7975(2) Å, *b* = 5.48433(5) Å, and *c* = 5.39562(5) Å)

*d*	*I*	*h*	*k*	*l*	*d*	*I*	*h*	*k*	*l*	*d*	*I*	*h*	*k*	*l*
11.3988270		2	0	0	4.44713	25	3	1	0	3.84626	66	0	1	1
3.79959	8	6	0	0	3.64438	57	2	1	1	3.50609	31	5	1	0
3.18818	20	4	1	1	2.84969	8	8	0	0	2.80026	22	7	1	0
2.74217251		0	2	0	2.70306999*		6	1	1	2.69781297		0	0	2
2.66611	6	2	2	0	2.62528	17	2	0	2	2.43064	23	1	2	1
2.32713	9	3	2	1	2.30657	5	3	1	2	2.28972298		8	1	1
2.27975	34	10	0	0	2.22357	68	6	2	0	2.19972	53	6	0	2
2.13811	11	5	1	2	1.97592	26	8	2	0	1.95913	36	8	0	2
1.95509	6	7	2	1	1.92313377		0	2	2	1.89979110		12	0	0
1.89633	49	2	2	2	1.82219	9	4	2	2	1.77740	6	3	3	0
1.75903	13	9	2	1	1.74129	8	10	0	2	1.73143	11	0	3	1
1.71586	13	6	2	2	1.70899	7	0	1	3	1.69681	21	5	3	0
1.69010	5	2	1	3	1.63698	9	4	1	3	1.62839	43	14	0	0
1.59414	14	7	3	0	1.58084	8	11	2	1	1.57556147		6	3	1
1.56163	55	12	2	0	1.55859182		6	1	3	1.55330	69	12	0	2
1.51006	5	1	3	2	1.49974	67	1	2	3M	1.49974	67	14	1	1M
1.48423	5	3	3	2	1.47971	74	8	3	1	1.46996	13	10	2	2
1.46563	69	8	1	3	1.43633	14	5	3	2	1.42484	6	16	0	0
1.40013	28	14	2	0	1.39412	23	14	0	2	1.37244	7	7	3	2
1.37108	26	0	4	0	1.36127	13	2	4	0	1.35153	75	12	2	2
1.34890	38	0	0	4	1.33956	9	2	0	4	1.33611	16	16	1	1
1.32660	6	1	4	1	1.28209	5	0	3	3	1.24273	44	14	2	2
1.22229	27	0	4	2	1.21532	6	2	4	2	1.21479	52	6	3	3
1.21039	28	0	2	4	1.20299	11	18	1	1	1.18620	11	14	3	1
1.17891	18	14	1	3	1.17675	7	9	4	1	1.16915	37	8	3	3M
1.16915	37	15	3	0M	1.16356	14	6	4	2	1.15328	12	6	2	4
1.13988	6	20	0	0	1.11178	9	12	4	0	1.09986	10	12	0	4
1.09438	8	16	1	3	1.09289	52	20	1	1	1.05256	5	20	2	0
1.05000	7	20	0	2	1.04882	19	14	4	0	1.03879	16	14	0	4
1.03429	26	6	5	1	1.02792	13	12	4	2	1.02223	5	18	3	1
1.02081	17	12	2	4	1.01996	40	6	1	5	1.01755	8	18	1	3
1.00734	5	14	3	3	1.00572	23	8	5	1	0.99252	19	8	1	5
0.99177	7	5	5	2	0.98057	7	20	2	2	0.97754	23	14	4	2
0.97142	24	14	2	4	0.96156	23	0	4	4	0.95816	5	2	4	4
0.95208	33	20	3	1	0.94829	31	20	1	3	0.90925	20	6	5	3
0.90269	33	6	3	5	0.89927	7	0	0	6	0.89055	5	19	4	1
0.88965	23	8	5	3	0.88870	8	6	6	0	0.88767	8	14	1	5
0.88608	5	22	1	3	0.88350	20	8	3	5	0.87683	8	26	0	0
0.87509	11	6	0	6	0.87064	6	20	0	4	0.86572	20	0	6	2
0.86323	16	2	6	2	0.85793	21	12	4	4	0.85463	57	26	1	1M
0.85463	57	0	2	6M	0.85190	57	2	2	6M	0.85190	57	20	3	3M
0.84908	6	9	6	1	0.84470	7	15	5	2	0.84408	6	6	6	2
0.84072	10	21	4	1	0.83995	7	12	5	3	0.83656	11	5	5	4M
0.83656	11	10	0	6M	0.83517	15	26	2	0	0.83389	14	26	0	2
0.83363	9	20	4	2	0.83026	5	24	1	3	0.82982	14	20	2	4
0.82918	6	23	3	2	0.82799	43	14	4	4	0.82646	7	11	6	1
0.82351	11	12	6	0M	0.82351	11	7	5	4M	0.82176	5	22	0	4

**Table 9:** X-Ray reference pattern for Sr_2_ErGaCu_2_O*_y_* (space group Ima2, *Z* = 4, *a* = 22.7964(3) Å, *b* = 5.47497(6) Å, and *c* = 5.38837(6) Å)

*d*	*I*	*h*	*k*	*l*	*d*	*I*	*h*	*k*	*l*	*d*	*I*	*h*	*k*	*l*
11.3982266		2	0	0	5.69911	15	4	0	0	5.32359	10	1	1	0
4.44207	29	3	1	0	3.84040	97	0	1	1	3.79940	9	6	0	0
3.63938	89	2	1	1	3.50354	28	5	1	0	3.18480	17	4	1	1
2.84955	38	8	0	0	2.79891	23	7	1	0	2.73749268		0	2	0
2.70096999*		6	1	1	2.69418297		0	0	2	2.66180	13	2	2	0
2.62194	18	2	0	2	2.42672	14	1	2	1	2.32368	7	3	2	1
2.30360	8	3	1	2	2.28841315		8	1	1	2.27964	13	10	0	0
2.22103	66	6	2	0	2.19772	56	6	0	2	2.13573	8	5	1	2
1.97412	36	8	2	0	1.95770	42	8	0	2	1.95301	6	7	2	1
1.92020345		0	2	2	1.89970	92	12	0	0	1.89352	50	2	2	2
1.81969	6	4	2	2	1.77453	7	3	3	0	1.75750	11	9	2	1
1.74026	6	10	0	2	1.72854	16	0	3	1	1.71377	17	6	2	2
1.70663	13	0	1	3	1.69430	15	5	3	0	1.68782	10	2	1	3
1.63490	7	4	1	3	1.62832	40	14	0	0	1.59222	16	8	2	2M
1.59222	16	7	3	0M	1.57971	8	11	2	1	1.57336190		6	3	1
1.56071	51	12	2	0	1.55679194		6	1	3	1.55256	57	12	0	2
1.49904	60	14	1	1M	1.49904	60	1	2	3M	1.48196	5	3	3	2
1.47789	70	8	3	1	1.46862	6	10	2	2	1.46413	76	8	1	3
1.43426	11	5	3	2	1.42478	6	16	0	0	1.39946	34	14	2	0
1.39357	32	14	0	2	1.37063	6	7	3	2	1.36874	32	0	4	0
1.35898	12	2	4	0	1.35048	68	12	2	2	1.34709	34	0	0	4
1.33778	10	2	0	4	1.33581	10	16	1	1	1.28013	6	0	3	3
1.24191	70	14	2	2	1.22029	24	0	4	2	1.21316	75	2	4	2M
1.21316	75	6	3	3M	1.20868	30	0	2	4	1.20275	12	18	1	1
1.18524	13	14	3	1	1.17811	18	14	1	3	1.17519	7	9	4	1
1.16771	34	8	3	3	1.16184	20	6	4	2	1.15180	12	6	2	4
1.11272	5	8	2	4	1.11052	8	12	4	0	1.09886	18	12	0	4
1.09271	44	20	1	1	1.07306	7	0	5	1	1.05225	8	20	2	0
1.04974	8	20	0	2	1.04775	21	14	4	0	1.03794	24	14	0	4
1.03267	26	6	5	1	1.02672	19	12	4	2	1.02160	7	18	3	1
1.01977	19	12	2	4	1.01867	42	6	1	5	1.01702	8	18	1	3
1.00637	7	14	3	3	1.00422	25	8	5	1	1.00042	8	22	1	1
0.99133	20	8	1	5	0.98015	12	20	2	2	0.97651	30	14	4	2
0.97052	25	14	2	4	0.96010	22	0	4	4	0.95671	5	2	4	4
0.95156	41	20	3	1	0.94786	41	20	1	3	0.93495	5	0	5	3
0.92796	6	0	3	5	0.91250	7	0	6	0	0.90787	31	6	5	3
0.90146	32	6	3	5	0.90032	5	18	3	3	0.89806	8	0	0	6
0.89600	5	14	5	1	0.88874	7	22	3	1	0.88835	24	8	5	3
0.88727	7	6	6	0	0.88681	10	14	1	5	0.88572	9	22	1	3
0.88235	21	8	3	5	0.87679	7	26	0	0	0.87398	9	6	0	6
0.87013	6	20	0	4	0.86427	27	0	6	2	0.86180	18	2	6	2
0.85688	26	12	4	4	0.85479	18	26	1	1	0.85332	38	0	2	6
0.85123	65	20	3	3M	0.85123	65	2	2	6M	0.84779	6	9	6	1
0.84372	5	15	5	2	0.84274	11	6	6	2	0.84012	7	21	4	1
0.83886	8	12	5	3	0.83500	19	26	2	0	0.83376	23	12	3	5M
0.83376	23	26	0	2M	0.83282	21	20	4	2M	0.83282	21	6	2	6M
0.82925	22	20	2	4	0.82704	59	14	4	4	0.82527	6	11	6	1
0.82253	13	12	6	0										

**Table 10:** X-Ray reference pattern for Sr_2_TmGaCu_2_O*_y_* (space group Ima2, *Z* = 4, *a* = 22.8024(3) Å, *b* = 5.46762(7) Å, and *c* = 5.38158(7) Å)

*d*	*I*	*h*	*k*	*l*	*d*	*I*	*h*	*k*	*l*	*d*	*I*	*h*	*k*	*l*
11.4012282		2	0	0	5.70060	18	4	0	0	4.43854	22	3	1	0
3.83541114		0	1	1	3.80040	17	6	0	0	3.63523	98	2	1	1
3.50216	31	5	1	0	3.18221	12	4	1	1	2.85030	72	8	0	0
2.79847	40	7	1	0	2.73381417		0	2	0	2.69958999*		6	1	1
2.69079391		0	0	2	2.65846	18	2	2	0	2.61884	31	2	0	2
2.46501	5	4	2	0	2.43334	5	4	0	2	2.42355	13	1	2	1
2.32094	6	3	2	1	2.28774337		8	1	1	2.28024	13	10	0	0
2.21927	79	6	2	0	2.19607	51	6	0	2	2.13372	12	5	1	2
1.97299	57	8	2	0	1.95745	50	10	1	1M	1.95745	50	8	0	2M
1.95154	5	7	2	1	1.93963	5	7	1	2	1.91771377		0	2	2
1.90020118		12	0	0	1.89114	57	2	2	2	1.81675	10	1	3	0
1.75651	12	9	2	1	1.75108	8	10	2	0	1.73961	7	10	0	2
1.72624	18	0	3	1	1.71208	24	6	2	2	1.70374	17	0	1	3M
1.70374	17	12	1	1M	1.69240	19	5	3	0	1.68574	18	2	1	3
1.65215	6	4	3	1	1.63304	12	4	1	3	1.62874	56	14	0	0
1.59077	23	8	2	2M	1.59077	23	7	3	0M	1.57908	9	11	2	1
1.57170189		6	3	1	1.56031	65	12	2	0	1.55522239		6	1	3
1.55218	72	12	0	2	1.49917	76	14	1	1	1.49657	14	1	2	3
1.48010	8	3	3	2	1.47655	85	8	3	1	1.46767	10	10	2	2
1.46286	73	8	1	3	1.43260	13	5	3	2	1.42515	9	16	0	0
1.39924	23	14	2	0	1.39337	23	14	0	2	1.36921	5	7	3	2
1.36691	30	0	4	0	1.35719	14	2	4	0	1.34979	70	12	2	2
1.34540	32	0	0	4	1.33600	18	2	0	4M	1.33600	18	16	1	1M
1.32261	5	1	4	1	1.28624	7	6	4	0	1.27818	13	0	3	3M
1.27818	13	12	3	1M	1.24142	58	14	2	2	1.21868	32	0	4	2
1.21174	73	2	4	2M	1.21174	73	6	3	3M	1.20713	39	0	2	4
1.20289	14	18	1	1	1.20042	7	2	2	4	1.18466	16	14	3	1
1.17756	23	14	1	3	1.16650	35	8	3	3	1.16047	17	6	4	2
1.15049	13	6	2	4	1.14387	5	16	2	2	1.11156	5	8	2	4
1.10963	9	12	4	0	1.09803	9	12	0	4	1.09289	64	20	1	1+
1.05228	9	20	2	0	1.04977	11	20	0	2	1.04704	28	14	4	0
1.03728	22	14	0	4	1.03141	31	6	5	1	1.02583	19	12	4	2
1.02130	6	18	3	1	1.01892	24	12	2	4	1.01740	60	6	1	5M
1.01740	60	18	1	3M	1.00566	9	14	3	3	1.00307	30	8	5	1
1.00058	11	22	1	1	0.99027	19	8	1	5	0.98896	7	5	5	2
0.98001	10	20	2	2	0.97577	33	14	4	2	0.96981	25	14	2	4
0.95885	47	0	4	4	0.95548	11	2	4	4	0.95135	36	20	3	1
0.94766	43	20	1	3	0.93361	11	0	5	3M	0.93361	11	12	5	1M
0.92972	6	6	4	4	0.90837	5	2	6	0	0.90675	23	6	5	3
0.90033	43	6	3	5+	0.89693	8	0	0	6	0.89523	6	14	5	1
0.88860	9	22	3	1	0.88732	30	8	5	3	0.88612	24	6	6	0M
0.88612	24	14	1	5M	0.88559	11	22	1	3	0.88135	25	8	3	5
0.87702	5	26	0	0	0.87295	8	6	0	6	0.86981	8	20	0	4
0.86312	20	0	6	2	0.86065	16	2	6	2	0.85604	28	12	4	4
0.85495	30	26	1	1	0.85223	36	0	2	6	0.85091	58	20	3	3
0.84986	14	2	2	6	0.84505	7	1	4	5	0.84168	15	6	6	2
0.83980	5	21	4	1	0.83801	12	12	5	3	0.83510	19	26	2	0
0.83384	16	26	0	2	0.83269	27	12	3	5M	0.83269	27	20	4	2M
0.83158	9	6	2	6	0.82887	27	20	2	4	0.82630	68	14	4	4
0.82438	6	11	6	1	0.82167	11	12	6	0					

**Table 11:** X-Ray reference pattern for Sr_2_YGaCu_2_O*_y_* (space group Ima2, *Z* = 4, *a* = 22.8113(2) Å, *b* = 5.48069(5) Å, and *c* = 5.39397(5) Å)

*d*	*I*	*h*	*k*	*l*	*d*	*I*	*h*	*k*	*l*	*d*	*I*	*h*		*k*	*l*
11.4057131		2	0	0	5.32904	5	1	1	0	4.44611	21		3	1	0
3.84441	25	0	1	1	3.64303	18	2	1	1	3.50639	26		5	1	0
3.18773	44	4	1	1	2.85142	11	8	0	0	2.80103	26		7	1	0
2.74035262		0	2	0	2.70325999*		6	1	1	2.69699308			0	0	2
2.62461	16	2	0	2	2.46998	8	4	2	0	2.42924	18		1	2	1
2.32602	7	3	2	1	2.30591	5	3	1	2	2.29022224			8	1	1
2.28113	53	10	0	0	2.22306	46	6	2	0	2.19972	34		6	0	2
2.13777	10	5	1	2	1.97582	22	8	2	0	1.95938	26		8	0	2
1.95478	6	7	2	1	1.92220339		0	2	2	1.90094	71		12	0	0
1.89547	30	2	2	2	1.82152	11	4	2	2	1.77635	8		3	3	0
1.75900	14	9	2	1	1.75320	8	10	2	0	1.74168	14		10	0	2
1.73035	8	0	3	1	1.71542	8	6	2	2	1.70841	5		0	1	3
1.69598	16	5	3	0	1.63655	9	4	1	3	1.62938	28		14	0	0
1.59356	11	7	3	0	1.58100	7	11	2	1	1.57490153			6	3	1
1.56193	56	12	2	0	1.55831191		6	1	3	1.55377	67		12	0	2
1.50017	57	14	1	1M	1.50017	57	1	2	3M	1.48348	6		3	3	2
1.47928	55	8	3	1	1.46992	26	10	2	2	1.46550	46		8	1	3
1.43570	15	5	3	2	1.40051	22	14	2	0	1.39462	18		14	0	2
1.37197	8	7	3	2	1.37017	29	0	4	0	1.36039	11		2	4	0
1.35162	64	12	2	2	1.34849	38	0	0	4	1.33917	5		2	0	4
1.33675	22	16	1	1	1.32575	8	1	4	1	1.24292	64		14	2	2
1.22157	26	0	4	2	1.21434	56	6	3	3	1.20993	33		0	2	4
1.20359	13	18	1	1	1.18623	11	14	3	1	1.17909	16		14	1	3
1.17631	6	9	4	1	1.16886	27	8	3	3	1.16301	11		6	4	2
1.15296	10	6	2	4	1.11153	7	12	4	0	1.09986	13		12	0	4
1.09462	9	16	1	3	1.09346	42	20	1	1	1.05300	6		20	2	0
1.05049	8	20	0	2	1.04867	26	14	4	0	1.03886	17		14	0	4
1.03372	25	6	5	1	1.02767	17	12	4	2	1.02072	24		12	2	4
1.01970	42	6	1	5	1.01783	7	18	1	3	1.00727	6		14	3	3
1.00522	23	8	5	1	1.00111	5	22	1	1	0.99232	17		8	1	5
0.99122	6	5	5	2	0.98089	9	20	2	2	0.97739	29		14	4	2
0.97140	24	14	2	4	0.96110	23	0	4	4	0.95771	5		2	4	4
0.95230	38	20	3	1	0.94859	36	20	1	3	0.90879	24		6	5	3
0.90238	30	6	3	5	0.89900	8	0	0	6	0.88925	21		8	5	3
0.88787	16	6	6	0M	0.88787	16	14	1	5M	0.88640	5		22	1	3
0.88324	17	8	3	5	0.87487	6	6	0	6	0.87084	7		20	0	4
0.86517	23	0	6	2	0.86269	15	2	6	2	0.85771	22		12	4	4
0.85537	24	26	1	1	0.85420	45	0	2	6	0.85196	60		20	3	3M
0.85196	60	2	2	6M	0.84987	6	16	1	5	0.84864	5		9	6	1
0.84450	7	15	5	2	0.84361	8	6	6	2	0.84080	8		21	4	1
0.83967	6	12	5	3	0.83617	6	5	5	4	0.83558	20		26	2	0
0.83432	17	26	0	2	0.83357	15	20	4	2M	0.83357	15		6	2	6M
0.82994	17	20	2	4	0.82782	52	14	4	4	0.82608	5		11	6	1
0.82333	8	12	6	0											

## Footnotes

1 Certain commercial equipment, instruments, or materials are identified in this paper to foster understanding. Such identification does not imply recommendation or endorsement by the National Institute of Standards and Technology, nor does it imply that the materials or equipment identified are necessarily the best available for the purpose.
